# Double-Barrel Shotgun: Probiotic Lactic Acid Bacteria with Antiviral Properties Modified to Serve as Vaccines

**DOI:** 10.3390/microorganisms9081565

**Published:** 2021-07-23

**Authors:** Leon M. T. Dicks, Matthew J. Grobbelaar

**Affiliations:** Department of Microbiology, University of Stellenbosch, Private Bag X1, Matieland 7602, South Africa; 20988257@sun.ac.za

**Keywords:** lactic acid bacteria, antiviral properties, vaccines

## Abstract

Contrary to the general belief that the sole function of probiotics is to keep intestinal microbiota in a balanced state and stimulate the host’s immune response, several studies have shown that certain strains of lactic acid bacteria (LAB) have direct and/or indirect antiviral properties. LAB can stimulate the innate antiviral immune defence system in their host, produce antiviral peptides, and release metabolites that prevent either viral replication or adhesion to cell surfaces. The SARS-CoV (COVID-19) pandemic shifted the world’s interest towards the development of vaccines against viral infections. It is hypothesised that the adherence of SARS-CoV spike proteins to the surface of *Bifidobacterium breve* could elicit an immune response in its host and trigger the production of antibodies. The question now remains as to whether probiotic LAB could be genetically modified to synthesize viral antigens and serve as vaccines—this concept and the role that LAB play in viral infection are explored in this review.

## 1. Introduction

Severe acute respiratory syndrome coronavirus (SARS-CoV) altered the world’s approach towards vaccine development. As of 16 March 2021, 264 vaccines have been engineered against SARS-CoV [1]. Of these candidate vaccines, 82 are currently undergoing clinical trials, whilst five of these trialling vaccines are in the fourth and final phase of trials [1]. Of the candidate vaccines undergoing clinical trials, nine are utilising mRNA as the vaccine platform. Two of these vaccines are the first to have been approved by the Food and Drug Administration (FDA) for human use [2].

Probiotics, described as “live microorganism(s) which, when administered in adequate amounts confers a health benefit on the host” [3,4], form an association with intestinal microorganisms and keep the gut microbiota in a balanced state [5]. Probiotics also stimulate the host’s immune system, interact with virus particles, compete with viral receptors for adhesion to receptors on epithelial cells, and regulate gut permeability [6]. This process must be tightly regulated. Gut microbiota in an imbalanced state can lead to dysbiosis and, as such, accentuate many intestinal-related illnesses such as diarrhoea and several forms of irritable bowel disease [7]. Certain strains of LAB can prevent or treat gastrointestinal infections such as necrotizing enterocolitis, acute infectious diarrhoea, antibiotic-associated diarrhoea, and infant colic [8]. Evidence of probiotic LAB playing a role in the prevention and treatment of acute respiratory tract infections has also been reported [9,10,11,12].

Although the composition of gut microbiota differs according to age, diet, medication, hormone levels, stress, and other environmental factors, a core group of autochthonous bacteria is always present [13]. A diet rich in fat and sugars supports the growth of *Bacteroidetes*, whereas a high-fiber diet shifts the balance towards *Firmicutes* [14]. A study conducted by Bolte et al. [15] showed a higher abundance of Firmicutes, *Ruminococcus* spp. of the *Blautia* genus, and the formation of endotoxins in individuals whose diet consisted of processed and animal-derived foods. In contrast, the authors found that a diet rich in nuts, oily fish, fruits, vegetables, cereals, and red wine supports the development of *Roseburia*, *Faecalibacterium*, and *Eubacterium* spp. Short-chain fatty acids (SCFAs) produced by these bacteria have anti-inflammatory properties [16]. The type of protein in the diet plays a role, as shown by Świątecka et al. [17]. In this study the authors showed that glycated pea protein increased cell numbers of *Bifidobacterium* and *Lactobacillus*, but to a lesser extent the proliferation of *Bacteroides fragilis* and *Clostridium perfringens.* Odamaki et al. [18] showed that *Actinobacteria*, *Bacteroidetes, Firmicutes*, and *Proteobacteria* are the predominant phyla in adults. Profound changes in the relative abundance of *Firmicutes* and *Proteobacteria* were, however, observed in the elderly [18]. Species of the genera *Lactobacillus* and *Bifidobacterium* form an integral part of the natural gut microbiome of humans and animals and their probiotic properties have been extensively studied [19,20]. The best-studied probiotic strains are from the genera *Lactobacillus*, *Lactococcus*, *Carnobacterium*, *Enterococcus*, *Streptococcus*, *Pediococcus*, *Propionibacterium*, and *Leuconostoc* [21,22]. To further understand the health benefits of many LAB species, Van Zyl et al. [23] summarized the beneficial effects of *Lactobacillus plantarum*, *Lactobacillus rhamnosus*, *Lactobacillus johnsonii*, *Lactobacillus casei*, *Lactobacillus salivarius*, *Lactobacillus reuteri*, *Lactobacillus acidophilus*, *Bifidobacterium infantis*, *Bifidobacterium breve*, *Bifidobacterium longum*, and *Bifidobacterium adolescentis*.

This review addresses (i) changes in gut wall permeability caused by viral infections and regulation thereof by LAB, (ii) modulation of the immune system, (iii) antiviral properties of LAB, and (iv) the possibilities of using LAB for vaccine production. One intriguing question is whether viral infections could be prevented by using probiotic LAB to block virus–target cell recognition sites or directly interact with virions. The mechanisms probiotic cells employ to inhibit the binding of an invading virus are discussed. The possibility of developing a probiotic to prevent or treat SARS-CoV related infections is addressed.

## 2. Regulation of Gut Wall Permeability

Permeability of the gut wall is tightly regulated to ensure that external pathogens and food-borne microorganisms do not cross the gut–blood barrier. Disturbances in the functioning of tight junction (TJ) proteins lead to an increase in gut wall permeability. Trans-cellular transport of molecules, proteins, and microbial cells across epithelial cells are regulated by TJ proteins, which consist of transmembrane proteins, occludin and claudin, the cytosolic protein, cingulin, protein Pals1 associated with Lin7, multi-PDZ domain protein 1 (MUPP1), and zona occludin transmembrane proteins connecting to the actin cytoskeleton [24,25]. Bacteria and viruses that cross the gut wall may cause bowel disorders such as irritable bowel syndrome (IBS), inflammatory bowel disease (IBD), and celiac disease [26,27,28]. Protein σ1 secreted by reoviruses allows for the internalisation of virus particles into TJs, rendering the gut wall and TJs ineffective [29]. Similar findings were reported for hepatitis C virus infections [30].

Several studies have shown that certain species of LAB could stabilise the gut wall. *L. rhamnosus* GG (LGG) stimulated the functioning of TJ proteins in pigs infected with rotavirus (RV) and alleviated symptoms of dysbiosis [31]. This suggested that LGG administration had the ability to reverse the negative effects of TJ, affecting viral infection by RV. In another study, patients with acute diarrhoea recovered quicker when administered LGG [32]. *Streptococcus thermophilus* and *L. acidophilus* induced the activity of occludin and zonula occludens 1 [33]. The authors ascribed the mode of activity to that of dephosphorylation of the myosin light chain (MLC) II, leading to activation of p38 and ERK pathways and stimulation of TJ proteins. *B. infantis* changed the permeability of epithelial cells by deactivating the epithelial layer pore forming protein claudin 2 [25]. Probiotics may also suppress the production of pro-inflammatory cytokines and, by doing so, prevent an increase in intestinal TJ permeability [25].

## 3. Immune System Modulation and Activation by Probiotics

The mucosal immune system plays a key role in the innate and systemic (adaptive) immune responses of humans and serves as first line of defence against antigens [34]. Upon the infection of a virus, the mucosal immune system prevents the entry and intracellular proliferation of virus particles. During viral infection, the innate immune system utilises apoptotic or phagocytotic mechanisms to degrade infected epithelial cells [35]. This is different from the immune response required to contain bacterial infections on the surface of epithelial cells, whereby the innate immune system would instead produce antibacterial compounds [36].

Several studies have shown that LAB can trigger an immune response that results in a rapid and efficient antiviral reaction [37,38]. A daily intake of heat-killed *L. plantarum* L-137 cells led to a significant decrease in upper respiratory tract infections by stimulating a Th1-type immune response in healthy adults with high physiological stress [37]. *Lactococcus lactis* JCM5805 displayed positive immunomodulatory effects on plasmacytoid dendritic cells (pDCs) in vitro, which decreased the morbidity attributed to the common cold [38]. Villena et al. [39] addressed the ability of probiotics to beneficially modulate IFN and inflammatory signalling pathways in IECs and immune cells, thus decreasing RV symptoms. A further example of immunoregulation and beneficial antiviral modulation of a human host was described by Tonetti et al. [40]. The authors reported that by nasally administering *L. rhamnosus* CRL1505 to mice that had been infected with influenza virus, the levels of influenza virus-specific IgA and IgG, as well as IFN-γ, in the serum and respiratory tract of infected mice increased significantly in comparison to the control group that did not receive strain CRL1505. Furthermore, the supplementation of mice with doses of non-viable *L. rhamnosus* CRL1505 displayed similar immunomodulatory effects to those reported with viable cells of *L. rhamnosus* CRL1505. This provides proof towards the beneficial use of paraprobiotics, defined as “inactivated microbial cells or cell fractions to confer a health benefit to the consumer” [41]. Probiotics with the ability to increase the immune response of individuals infected by viruses are referred to as “immunobiotics” [37,38]. The role immunobiotic LAB play in viral infections and the host’s immune system, cells, structures, and components are summarised in Table 1. Examples listed in the table have been chosen based on their general presence in the immune system and relevance to this review.

DCs are critical to the activation and corrective functioning of the innate immune system. This is due to DCs displaying phagocytotic capabilities as well as being able to produce type-I IFNs (IFN-α and IFN-β). IFNs serve as the first line of viral defence by blocking viral replication [47]. According to Kanauchi et al. [48], the majority of microbial specimens used in their study showed insignificant IFN upregulation. However, the study did elucidate *Lactococcus lactis* strains that showed significant modulation of DCs by directly stimulating DC cells to produce type-1 and -3 IFNs (IFN-α, -β and -γ) [48].

*L. lactis* induces TLR9/MyD88 signalling and IFN when engulfed by DCs [49]. The DNA of *L. lactis* acts as a ligand that attaches to TLRs and activates IFN-α production. Although direct DC stimulation by *L. lactis* is considered the most probable antiviral mechanism [48], some strains activate NK cells in vivo and in vitro, leading to the production of IFN-α [50]. *L. lactis* subsp. lactis JCM5805 induced the cytotoxicity of NK cells, contributing to host defence against infection by parainfluenza virus particles [50]. *L. lactis* subsp. lactis JCM5805 increased the levels of IFN-α induced DCs, which in turn increased the cytotoxicity levels of NK cells [50].

**Figure 1:**
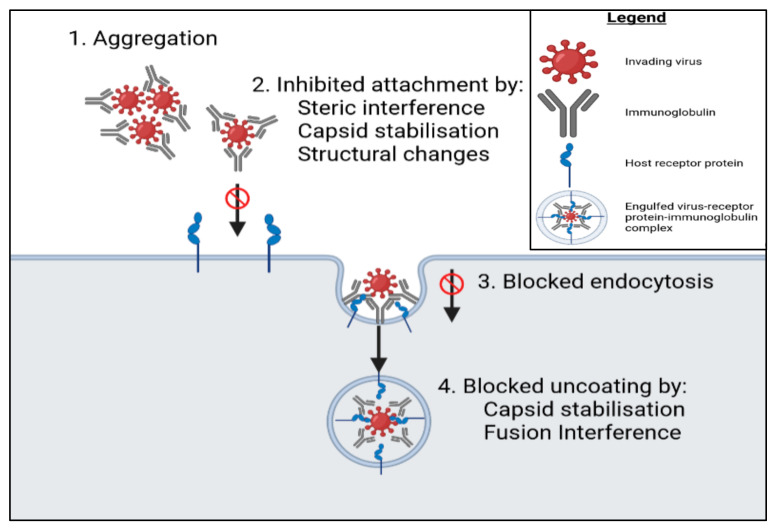
Suggested mechanisms of immunoglobulin-mediated blocking of viral replication. There are 4 possible ways antibody binding can prevent the eventual nucleic acid release of the infecting virus. (**1**) By inducing viral aggregation, mucosal antibodies obstruct the movement of virions through mucous, which reduces penetration through epithelial cells, reduces viral access to susceptible mucosal CD4 T cells and dendritic cells, and prevents transcytosis [56]. (**2**) Antibody detection of infecting virions can, based on the 3 mentioned techniques, result in the inability for these virions to bind to receptor proteins on susceptible cells, in turn blocking the attachment of the virions to host cells. (**3**) Transmembrane receptors are readily endocytosed, which is beneficial to an infecting virion. However, antibodies have the capacity to target these transmembrane receptors, subsequently limiting endocytosis of the receptor-virion complex [57]. (**4**) By utilising the 2 techniques mentioned, antibodies may block release of the infecting virus’ genome.

A review by Kitazawa and Villena [51] commented on the immunomodulatory effects probiotics have in treatment of viral infections and focused on *L. rhamnosus* CRL1505 in the treatment of respiratory syncytial virus (RSV) infections. Recent research has shown that immunobiotics could be used in the prophylactic treatment of respiratory viral infections [51]. Yasui et al. [52] showed that orally administered immunobiotics had a stimulatory effect on the mucosal and the anti-viral humoral immune systems. *B. breve* YIT4064 augmented the production of anti-viral antibodies by directly activating B cells to produce antibodies [52]. The immunoglobulins included those that identified polio, influenza, and rotaviruses. The resulting antibodies neutralise an infecting virion by blocking the glycoprotein spikes used by the virus to bind to a host target cell [53]. This binding subsequently interferes with target cell viral uptake, in turn diminishing the ability of the virus to replicate [54]. Figure 1 illustrates the mechanisms utilised by immunoglobulins to interrupt viral cell uptake by a host [55].

**Figure 2:**
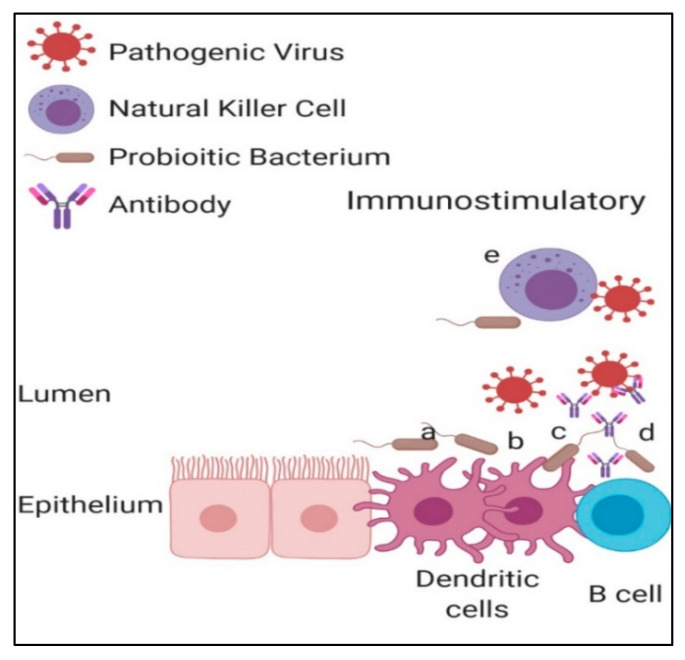
Immunomodulatory effects offered by probiotics to enhance the inhibition of viral shedding. To confer an immunomodulatory effect on its host, probiotics can either elicit immuno-stimulatory or -repressive effects. Mechanisms (**a**,**b**) represent direct DC activation by probiotic EPS and DNA identification, respectively. The technique represented by (**c**) also represents the enhanced DC activation upon identification of probiotic factors; however, this technique requires the DC engulfment of a probiotic. Once activated, the DCs produce pro-inflammatory cytokines which initiate further activation of innate and acquired immune response systems. Enhanced immunoglobulin production initiated by the recognition of probiotics by B cells is represented by the letters (**d**,**e**), showing the increased cytotoxicity attributed to NK cells upon their recognition of probiotic bacteria.

Another way by which LAB can communicate with their host’s immune and non-immune cells is via the production of immunomodulatory extracellular polysaccharides (EPSs). Laiño et al. [39] reported that *Lactobacillus delbrueckii* OLL1073R-1 produced EPSs that interacted with PRRs on the surface of host immune cells which proved to induce antiviral immune responses in the host. Whilst the previously discussed mechanisms of probiotic immunomodulation are all stimulatory, some probiotics can induce immunosuppressive effects in their host. The result of this is the lowered or diminished production of pro-inflammatory cytokines, and thus, inflammation [58]. Although inflammation is beneficial as it recruits antiviral compounds to the site of infection, an excessive inflammatory response can lead to collateral damage of host tissue [59] and an increase in number of cells infected by an untreated virus. Live and heat-attenuated *L. plantarum* and *L. reuteri* are examples of LAB that were found to have immunosuppressive qualities when intranasally administered to wild-type mice [60]. The treatment of mice with these probiotics led to the complete protection of these mice from pneumonia virus lethal infection. The authors speculate that the reason for this beneficial result was that these LAB diminished granulocyte and pro-inflammatory cytokine expression owing to the transcriptional regulatory capabilities of their host. Tonetti et al. [40], whose study was documented earlier in this review, also found that mice nasally treated with *L. rhamnosus* CRL1505 had reduced levels of interleukin-17 and increased levels of interleukin-10 during influenza infection. This aided in the protection of mice from influenza infection without having to induce inflammatory-mediated lung damage. Figure 2 shows the mechanisms in which immunoregulatory probiotics have been shown to function as viral replication inhibitors. 

It is evident that one of the ways to best exploit the immunomodulatory effects associated with probiotics is to accentuate probiotic ligand binding to the PRRs of DCs such as TLRs. Depending on the LAB species used, this will in turn, increase cytokine production when necessary and increase the mucosal immune system’s inflammatory response. In doing so, the number and activity of host-produced antiviral molecules, cells, and peptides will increase, leading to the more rapid clearance of the infecting virus from the host’s body. In contrast, the suppression of the immune system by LAB can also manifest a beneficial response, especially when the host is suffering from hyperinflammation.

## 4. Production of Antiviral Substances and Direct Virus Interaction by Probiotics

The most commonly reported mechanism of viral inactivation is direct virus–probiotic interaction [6]. An example of such a mechanism was reported by Botić et al. [61], who found that LAB probiotics could trap vesicular stomatitis virus (VSV) within their cells. The beneficial LAB that are capable of viral entrapment were *Lactobacillus paracasei* A14, *L. paracasei* F19, *L. paracasei/rhamnosus* Q8, *L. plantarum* M1.1, and *L. reuteri* DSM12246. Engulfment of the VSV virions was achieved by the respective LAB interacting with and identifying the envelope of the virus. An in vivo study conducted by Wang et al. [62] discovered that *Enterococcus faecium* NCIMB10415 was able to absorb virions of swine influenza A virus (SwIV) (a respiratory virus affecting pigs). Furthermore, the mechanism of action attributed to *E. faecium* NCIMB10415 was found to be twofold. Not only could the bacteria engulf the virus but upon absorption, the bacteria also activated the host cell’s innate immune response, thus allowing the activated epithelial cells to elicit a pro-inflammatory response to counter the viral infection. This immunoactivation is mediated by the bacterial release of molecules that bind and activate PRRs to initiate IFN production. Another study focusing on the direct inactivation by LAB documented *Lactobacillus gasseri* CMUL57′s ability to inhibit the enveloped virus herpes simplex type 2 (HSV-2). Although the mode of action was similar to those mentioned above (inactivation-by-trapping) [63], an important criterion that a probiotic must meet is an ability to survive in normally inhospitable environments [8]. The fact that *L. gasseri* CMUL57 was isolated from the vaginas of Northern Lebanese women shows the extreme adaptive qualities this probiotic strain possesses.

*L. lactis* JCM5805 was previously documented to display immunomodulatory activity in the form of direct activation of DCs or NK cells [50]. In this study *L. lactis* was used as a model probiotic and as such does not represent the mode of action of all probiotics. Salminen et al. [64] unsurprisingly found contradictory results. Their results indicated that probiotic bacteria such as LGG and *Bifidobacterium lactis* Bb-12 (now reclassified as *Bifidobacterium animalis* Bb-12) could bind to two RVs: Nebraska calf diarrhoea virus (NCDV), and human rotavirus strain Wa. This binding caused the inactivation of these viruses, which was quantified by the reduced shedding of these viruses. Throughout literature, the mechanisms and capabilities ascribed to a probiotic are strain-dependent. Therefore, the results reported by Salminen et al. [64] do not disprove the study performed by Kanauchi et al. [48] but rather indicate that the different strains used in these studies have contrasting modes of helping a host overcome the pathogenesis attributed to viral infection.

Metabolites and compounds produced by probiotics can also have antiviral properties. An example of such is hydrogen peroxide (H_2_O_2_). H_2_O_2_ production is a common trait of many *Lactobacillus* species as these bacteria lack haem and as such cannot utilise the cytochrome system. Consequently, these bacteria have to make use of flavoproteins which convert oxygen to H_2_O_2_ as opposed to water—a trait of haem-containing bacteria [65]. H_2_O_2_ has been shown to be toxic to viruses such as HIV type I (HIV-1) and HSV-2 [66]. In an independent study to that of Eschenbach et al. [65], *L. gasseri* ATCC33323 was found to inhibit 68% of HIV-1 replication, whilst *Lactobacillus crispatus* ATCC33820 could inhibit 50% of HSV-2 replication [67]. The low pH of lactic acid produced by LAB (pH 3.51) interferes with the external integrity of the envelopes from murine norovirus [68], influenza virus [69], HIV-1, and HSV-2 [66].

**Figure 3:**
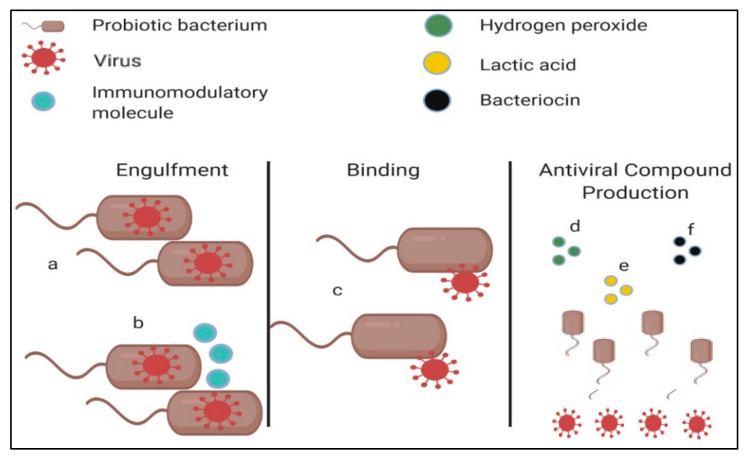
Various means by which probiotic bacteria have been adjudged to directly inactivate viruses. The first mechanism of viral inactivation by probiotics requires the engulfment of viral particles by probiotics (**a**,**b**). Some strains can engulf virions whilst also producing molecules that can initiate the activation of the innate immune system (**b**). As opposed to viral engulfment, some LAB can inactive viruses by merely binding to them (**c**). Antiviral compounds produced by probiotics have also been studied. These compounds include hydrogen peroxide (**d**), lactic acid (**e**), and bacteriocins (**f**), which initiate the formation of viral aggregation.

Unlike H_2_O_2_ and lactic acid, some antiviral compounds produced by probiotics are ribosomally produced. Although bacteriocins are ribosomally post-translated peptides that have antibacterial activity against species closely related to that of the bacteriocin-producing strain [70], these compounds have also been found to contain antiviral properties. Such bacteriocins include staphylococcin 188, enterocin AAR-71, enterocin AAR-74, and erwiniocin NA4. The antiviral activity of these bacteriocins was studied against coliphage Hsa and the results were highly promising. Both enterocin AAR-74 and staphylococcin 188 were found to reduce viral progeny tenfold, whilst the results obtained from enterocin AAR-71 and erwiniocin NA4 were even more exemplary. These two bacteriocins completely abolished any viral progeny [71]. In an independent study, staphylococcin 188 inhibited influenza A and Newcastle disease virus (NDV) in in vivo and in vitro models [72]. Todorov et al. [73] concluded that *E. faecium* ST5Ha isolated from smoked salmon could produce pediocin-like bacteriocins which were found to have activity against the influenza A virus as well as HSV-1. A bacteriocin’s antibacterial mechanisms are well studied and understood; however, the compound’s antiviral ability is less well deciphered. Only one antiviral mechanism has been described until now, that being the ability of bacteriocins to form viral aggregations. The result of this is that the viral receptor sites on host cells would be blocked, thus making it impossible for any viruses, other than the ones already attached to the host cell, to bind and infect host cells [74]. This conclusion was made when it was found that Enterocin CRL35 could inhibit the late stages of HSV-1 and HSV-2 replication. Interestingly, exceptionally low concentrations of the bacteriocin were necessary to ensure the replication inhibition of the viruses (the CC_50_ value of the bacteriocin was lower than 1200 μg/mL). In Figure 3, the various mechanisms of direct viral inhibition by LAB can be seen.

Thus far, direct virus–probiotic interactions and antiviral compounds produced by probiotics are seemingly the most plausible virus prophylactics; however, there are currently no pharmaceutical products of this sort available on the market.

## 5. Virus–Probiotic Viral Receptor Competition

Specific proteins on the capsid or envelope of an invading virus bind to receptor proteins on the cell membrane of a target cell in the event of infection. This process occurs irrespective of the virus type and is necessary for the subsequent viral entry into the host cell [75]. This therefore poses the idea that if the viral identification and binding of cellular receptor proteins could be interfered with, viral infections could be eradicated. There are four well documented ways in which probiotics can inhibit the binding of an invading virus, as discussed below.

### 5.1. Binding of Proteins to Cell Membrane Receptors

Intra- and extracellular compounds produced by microorganisms may have antiviral properties, as was proven in *L. casei, Lactobacillus fermentum, B. adolescentis*, and *Bifidobacterium bifidum*-based experiments [76]. Strains from these species decreased the rate at which RVs adhered to MA104 cells (epithelial kidney cells derived from African Green monkeys), as proteins derived from these experimental strains were found to bind to receptors Hsc70 and β3-integrin located in the membrane of MA104 cells [76].

### 5.2. Adhesion of Bacterial Cells to Virus Particles

Salminen et al. [64] showed that LGG and *B. lactis* could adhere to and subsequently inactivate RVs. The adhesion resulted in the blocking and saturation of spike proteins on the surface of virus particles, which in turn prevented RV infection in an animal host. The study conducted by Salminen et al. [64] was performed using calf and human cell lines.

### 5.3. Probiotic Biofilm Formation

Biofilms may form when probiotics adhere to epithelial cells [77]. Whilst this epithelial binding offers a survival advantage to the administered probiotics, the resulting probiotic biofilm is also extremely favourable to humans. Probiotic biofilm formation on epithelial cells results in the viral receptors of the cells within the epithelial layer becoming completely covered by a mat-like biofilm, hence being unreachable for invading viruses [6]. This in turn limits the viral entry and infection of human cells.

### 5.4. Probiotic-Mediated Compositional Modulation of the Gut, Lung, and Respiratory Tract Microflora

Histo-blood group antigens (HBGAs) are similar to the ABO antigens found in human red cells; however, they are found in different bodily tissues and fluids to ABOs [78]. These HBGAs play an important role in viral pathogenesis, as specific viruses bind to carbohydrates of the HBG family [79]. Viruses and pathogenic bacteria bind to HBGAs in their first step of pathogenesis, as these antigens provide an attachment receptor for these pathogens [6]. However, the production of these HBGAs is the result of a functional *FUT2* gene in the host. If this gene is instead a dysfunctional artefact, the host would not be able to encode these receptor factors [80]. Problematically, however, gram negative commensal bacteria can display these antigens and act as secondary receptors in human tissue [81]. A simple way of overcoming this issue would be to ensure that the gut, respiratory tract, and lung epithelia are colonised by gram-positive bacteria. By administering gram-positive probiotics, the composition of the host’s microflora will shift, and optimally, the gram-positive bacteria that are being administered should create an exclusive environment to ensure that they (the most beneficial microorganisms) are the primary occupants of the host.

**Figure 4:**
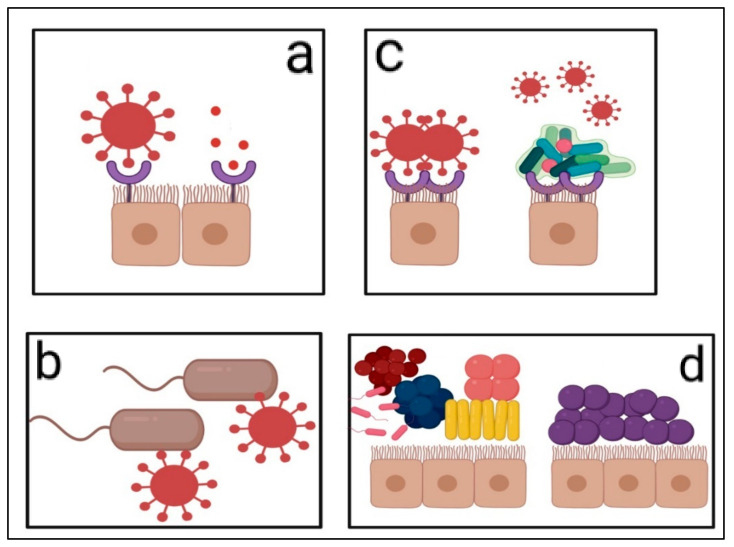
Probiotic-based interference with epithelial virus receptors. (**a**) Some probiotics produce proteins that mimic viral binding proteins, therefore saturating epithelial receptors [76]. The red circles represent probiotic-derived proteins that mimic proteinaceous spikes of infecting virions as seen on the left-hand side of (**a**). (**b**) Other probiotics can occupy viral spike proteins upon binding to an infecting virus. This results in the inability of the virus to detect receptor proteins of host cells [64]. (**c**) Probiotic biofilm formation, as seen on the right-hand side, covers the receptor proteins of epithelial cells, rendering invading viruses incapable of attaching to host cell receptor proteins. This results in the inability of the virus to infect a susceptible host as seen on the left-hand side of Figure (**c**). Where the mechanism depicted by (**b**) represents probiotic hindering of viral spike proteins, (**c**) represents the incapacitation of host cell receptor proteins to be detected by viruses due to the fact that these proteins are covered by probiotic biofilms [6,77]. (**d**) In the Figure to the left-hand side of (**d**), a vast array of non-synonymous bacteria colonises the epithelial cells of a host. By ensuring that these cells are rather solely colonised by gram-positive bacteria, as seen on the right-hand side of (**d**), the ill effects of gram negative, non-advantageous bacteria can be eradicated by flooding the gut with probiotic gram-positive bacteria [6,81]. These negative effects include secondary viral receptor production which can lead to the infection of a host, even if its own receptors are incapacitated by one of the mechanisms shown in (**a**–**c**). Further, Table 2 offers additional examples of instances where LAB have displayed immunomodulating, gut wall enhancing, antiviral capabilities.

The administration of optimal probiotics can therefore be beneficial to the host as their presence and biofilm formation can inhibit the associations required for the virus to infect its human or animal target. The four different methods of viral receptor interference can be visualised in Figure 4.

## 6. SARS-CoV Probiotic-Based Vaccine and Secondary Symptom Treatment Possibilities

This review has focussed on the immunomodulatory, supplementary, and prophylactic capabilities of LAB during host viral infections. However, one of the most plausible approach in which probiotic bacteria can be used to combat viral infections, pandemics, and outbreaks is by genetically modifying LAB strains to act as vaccines. One of the reasons probiotics are suitable for this is because to be classified as a probiotic, a bacterial strain has to meet criteria that judge a strain by the health advantages it conveys to its host as well as how well the candidate can survive in a human system [90]. Therefore, the chosen probiotic will already be known to not cause any harm or pathogenesis towards its host if the host being administered the probiotic is not immunocompromised. If the probiotic recipient is immunocompromised, suffering from certain clinical conditions, or is recovering post-organ transplant, the administration of probiotics may do more harm than good, as the probiotic LAB may exploit the weak immunity of their host and emerge into opportunistic pathogens [91]. If immunocompromised, investigating the possibility of using paraprobiotics as the basis of the probiotic-based vaccine may be beneficial. As previously mentioned, there are no probiotic-based vaccines currently in the pipeline to be used as COVID-19 vaccines, but multiple research groups, both independent and university-based, have started conducting research into the possibility of genetically modifying probiotic bacteria to code for the presentation of SARS-CoV spike proteins on their capsules [92]. By doing so, the body would initiate an innate immune response to the non-pathogenic manipulated bacteria. This will allow for the body to produce antibodies that will uniquely recognise the spike proteins of SARS-CoV, in turn preparing the body’s acquired immunity for the production of antibodies against incoming coronavirus virions [93]. Developing an efficacious probiotic-based COVID-19 vaccine may not be that farfetched. Viable cells of *L. lactis* strains presenting with genetically modified induced cytoplasmic RV spike-protein subunit VP8* were administered to mice, resulting in the development of IgA antibodies within the treated mice [94]. In this same study, mice that were administered a genetically modified strain of *L. lactis* with the VP8* subunit protein anchored to its cell wall showed the formation of intestinal and systemic anti-RV antibodies. Cytoplasmic and cell wall-displaying recombinant strains of *L. lactis* prevented RV infection by 50% and 100%, respectively [94].

Although no probiotic-based vaccines are currently being produced or entered into clinical trials, 4D Pharma in the United Kingdom has entered phase-II clinical trials with a strain of *B. breve* known as MRx-4DP0004 that they hope can suppress the “inflammatory storm” brought upon patients suffering from COVID-19 [95]. Evidence suggests that the triggering of hyperinflammation by the SARS-CoV virus plays a vital role in the mortality attributed to this virus [96]. The immunomodulatory properties attributed to the *B. breve* strain in question are described as being capable of “targeted immunomodulation rather than broad immunosuppression” [92], and as such are expected to reduce the levels of neutrophils and eosinophils, which should result in lower than normal amounts of T cells, DCs, and pro-inflammatory cytokines. This probiotic has shown in its preclinical trials to have the ability to downregulate the hyperinflammatory response elicited by the SARS-CoV virus whilst still maintaining the necessary antiviral response, in addition to not inhibiting any other necessary inflammatory responses needed to treat other infections [95]. The aim of having this probiotic medication accepted for general use is to prevent coronavirus-associated symptoms from progressing from mild/moderate to severe. This would lower the strain placed upon hospitals in terms of the outnumbering of patients with respect to beds, ventilators, IC units, and medical staff.

Hypothetically, by expressing the spike protein in *B. breve* YIT4064 one might enjoy favourable results. This hypothesis is based on the ability of this probiotic to “augment the production of anti-viral antibodies” [52]. Therefore, these manipulated bacteria could initiate immunoglobulin production by presenting with its genetically engineered spike proteins while increasing the production of immunoglobulins. This would offer a twofold advantage over any other vaccine for viruses currently available. Although the nucleic acid sequence that encodes the coronavirus spike proteins is easy to identify, isolate, synthesise, and introduce into a bacterium, the aggregation of this protein is a hurdle that researchers are finding difficult to overcome. The spike protein of the SARS-CoV virus, like that of influenza and hepatitis C viruses (among others), is glycosylated, and as such has carbohydrate molecules attached to the distal end of the protein [97]. The glycosylation of this protein is also what made modelling the structure of the protein difficult in primary descriptive studies of the novel coronavirus.

A review written by Baud et al. [98] accumulated clinical data demonstrating the ability of probiotics to prevent the contraction of COVID-19. The article focusses on general studies of probiotic antiviral abilities. In particular, LAB probiotic species such as *L. plantarum* and *B. bifidum* are mentioned for their immunomodulatory properties administered in response to viral infection as well as their modulation of TJ functioning in the gut epithelial barrier. The latter is, according to the study, an important characteristic of probiotics that should be considered, because samples of the SARS-CoV virus have been isolated from the gut and stool of infected patients. Furthermore, the article states that although mechanistically probiotics should primarily affect the coronavirus from an immunomodulatory point of view, the antiviral properties of probiotics should not be ignored. It could therefore be proposed that a bacteriocin-producing probiotic such as *Staphylococcus aureus* AB188 be studied. *S. aureus* AB188 produces the bacteriocin staphylococcin 188 which, according to Saeed et al. [72], was found to be active against influenza A and NDV. A study comparing the anti-coronavirus capacity and activity of a dosage of live *S. aureus* AB188 cultures with a dosage of isolated staphylococcin 188 should thus be conducted. Although other bacteriocins such as enterocins were found to be active against viruses, staphylococcin 188′s ability to inactivate respiratory virions of the SARS-CoV virus type (such as influenza and NDV) may prove beneficial in creating a novel coronavirus prophylactic.

## 7. Conclusions

The combination of an increased risk of virus contraction due to global warming and increased human geographical movement [48], with the emerging resistance of viruses towards antiviral agents due to their ability to rapidly mutate [66], has resulted in a call from society to pharmaceutical manufacturers and virologists alike to produce new, more effective vaccines and viral prophylactics. This problem has been exacerbated by the current coronavirus pandemic sweeping the globe. The prevalence of this deadly, novel, respiratory virus has led researchers to try and find a viable vaccine that can be globally administered. 

Whilst the treatment and prevention of the novel coronavirus are at the forefront of current virological research, other viruses are still without cures or vaccines. It should be noted that since the 1990s probiotics have been studied for their antiviral capabilities and could be possible novel antiviral treatments. The key attributes probiotics portray that could facilitate their exploitation for preventative and prophylactic treatment of viral infections are their immunomodulatory, gut epithelial barrier-modulating, antiviral agent-producing, and viral receptor-interfering capabilities. However, despite a plethora of research being conducted in the probiotic–virus field, no probiotic-based antivirals or vaccines are available on the market. Of the antiviral probiotic qualities previously mentioned, it could be argued that the antiviral agents produced by LAB as well as these species’ ability to inhibit target cell viral binding should have been encouraging enough to justify the production of a probiotic-centred antiviral, although one is yet to be produced. It is exciting, however, to see that a possible *B. breve*-based medication is currently undergoing a clinical trial to evaluate its ability to decrease the hyperinflammatory response triggered by COVID-19 infection. Along with this treatment, two other streams of probiotic based coronavirus antivirals should be investigated further, these being a vaccine consisting of SARS-CoV spike protein-displaying *B. breve* YIT4064 bacteria as well as a *S. aureus* AB188 or staphylococcin 188-based viral prophylactic.

This review illustrates that there is a capacity for probiotics and their compounds to be considered for their individual medicinal purposes. This is especially the case if the probiotics are used to interfere with virus–target cell recognition or directly interact with virions, making viral infection impossible. It is also important to remember that the functions of all probiotics are strain-dependent and that multiple studies must be conducted to ensure that a specific strain can elicit a desired antiviral affect without causing its host any harm.

## Figures and Tables

**Table 1:** Viral- and host-produced compounds modulated by immunobiotic LAB. Information obtained from references [42,43,44,45,46].

Immune System Component:	Examples:	Function:
Pathogen-associated molecular patterns (PAMPs)	DNA, RNA, surface glycoproteins	Act as ligands belonging to the virus in the form of conserved sequences or structures. Recognition molecules of the host identify the ligands and triggers the appropriate immune response.
Damage-associated molecular patterns (DAMPs)	Molecules such as ATP, DNA, hyaluronan fragments, and the chromatin-associated leaderless secreted protein HMGB1 secreted from damaged host cells	Unlike PAMPs, DAMPs originate from virus-infected host cells. DAMPs are specific in sequence and structure.
Pattern recognition sequences (PRRs)	Toll-like receptors (TLRs), nucleotide-binding oligomerization domain (NOD)-like receptors (NLRs)	PRRs are sensors mainly encoded by cells of the innate immune system (e.g., DCs, macrophages, neutrophils, etc.) that detect PAMPs and DAMPs. This initiates the release of cytokines and leads to an antigen-specific systemic immune response.
Cytokines	Interferons (IFNs) such as type 1 IFN-α/β and IFN-γ, interleukins IL-1, 2, 6, 8, 10, 15, and 18, tumour necrosis factor (TNF), cytokines TNF-α, and chemokines such as CXCL-8	Cytokines are small signalling proteins, peptides, or glycoproteins that play an integral part in inflammation and immunity regulation. Type-1 IFNs are the only cytokines solely associated with viral immunity, as opposed to also being involved in bacterial immunity.
Macrophages		Employ phagocytosis to “digest” viral particles. While doing so, macrophages release cytokines of their own upon PAMP recognition by their PRRs.
Granulocytes	Neutrophils, eosinophils, and basophils	Neutrophils are the most prominent white blood cells in the human body. These phagocytotic cells have a short half-life, followed by apoptosis. A major chemoattractant of neutrophils are chemokines, particularly CXCL-8.
Dendritic Cells	Nasal, epidermal, intestinal, pulmonary, tracheal, etc. Dendritic cells are external cells that come into contact with the external environment	Initiate both innate and system immune responses. They too are phagocytotic cells and can thus engulf viral particles. Furthermore, once infected by a virus, DCs initiate a T-cell response by displaying viral antigens on the type-I major histocompatibility complex (MHC). If the DC has only phagocytotically engulfed the virus, it can display the associated antigens on a type-II MHC which will elicit a systemic immune response. DCs can also produce cytokines such as type-I IFNs when their TLRs bind to viral particles such as RNA.
T-cells	Cytotoxic T-cells (T_C_ cells) such as CD8 and T8, and T-helper cells (T_H_ cells)	T_H_ cells initiate B cells to produce antibodies and in turn are extremely important in the systemic immune response, whilst T_C_ cells, such as CD8-T cells in particular, form pores in infected cells after which cytotoxins are released by the cells killing the infected cell as well as any viruses inside it.
Natural killer (NK) cells		NK cells chemotoxically kill infected cells and their infiltrating viruses. However, NK- and CD8- T cells recognise these infected cells differently.
Peripheral blood mononuclear cells	Any blood cell with a circular nucleus such as T cells, B cells, and NK cells	Engulf and phagocytose viruses whilst releasing pro-inflammatory cytokines

**Table 2:** Viral- and host-produced compounds modulated by immunobiotic LAB.

Mechanism of Action:	Probiotic Strain:	Studied Virus:	Test Patients /Tissue:	Result:	Reference:
Immunoregulation	*L. delbrueckii* ssp. bulgaricus OLL1073R-1	Common cold symptoms	Elderly people	A significant increase in NK cell cytotoxicity, resulting in a reduced risk of ailing from cold symptoms.	[82]
	*L. paracasei* ssp. paracasei, *L*. *casei* 431	Influenza	Healthy adults with influenza vaccination	A significant increase in influenza-specific IgG, IgG1, and IgG3 in plasma and IgA in saliva.	[83]
	*B. animalis* (Bb12)	Polio and rotavirus	Healthy 6-week-old infants	A significant increase in polio and rotavirus-specific IgA antibodies.	[84]
	*L. lactis* JCM5805	Common cold	Healthy adults	Activation of pDCs amongst peripheral blood mononuclear cells (PBMCs) and as such, a significant reduction in morbidity attributed to the common cold.	[38]
		Influenza	Healthy adults	A significant increase in IFN-α mRNA in PBMCs, meaning a significant decrease in the number of days ailing from influenza symptoms such as sore throats and coughs.	[12]
Tight junction maintenance and functional improvement	*L. reuteri* LR1		Intestinal porcine epithelial cells	MLCK-dependent dephosphorylation of TJ subunit proteins such as ZO-1 and occluding resulting in a decreased pathogen flooding of the lamina propria	[85]
	*B. longum* and LGG lysates		Normal human epidermal keratinocytes	A lysate-induced increase in claudin 1 levels in keratinocytes correlating with the decreased pathogen flooding of the lamina propria.	[86]
Direct virus inactivation by probiotics/probiotic compounds	*Lactobacillus brevis*	Herpes simplex virus 2	Vero cells	Cell wall interaction with virus envelope resulting in reduced viral replication	[87]
		Herpes simplex virus 2	Vero cells	No proteinaceous heat resistant proteins isolated from *L. brevis* extract interacted with HSV2 envelopes.	[87]
	*L. plantarum* PCA236	RV and transmissible gastroenteritis virus (TGSV)	Human and animal intestinal and macrophage cell lines	Reactive oxygen species (ROS), nitric oxide (NO^-^), and H_2_O_2_ interaction with RV and TGSV virion envelopes.	[88]
Epithelial cell virus receptor interference	*L. casei* DN114 001 and *Bacteroides thetaiotaomicron*	RV	Human epithelial cells	Soluble compound production binding to viral receptors, resulting in glycosylation and thus structural isomerisation of the receptor, making it unable to attach and identify RV virions.	[89]

## Data Availability

Not applicable.
